# Increased CD16a (FcγRIIIA) Expression in The Tumor Microenvironment of Atypical Neurofibromatous Neoplasms of Uncertain Biologic Potential May Be Associated with Progression from Neurofibromas to Atypical Neurofibromas

**DOI:** 10.3390/jpm13121720

**Published:** 2023-12-17

**Authors:** Min-Kyung Yeo, Yeong Jun Koh, Jong-Il Park, Kyung-Hee Kim

**Affiliations:** 1 Department of Pathology, Chungnam National University School of Medicine, Munwha-ro 266, Daejeon 35015, Republic of Korea; mkyeo83@cnuh.co.kr; 2 Department of Computer Science & Engineering, Chungnam National University, Daejeon 34134, Republic of Korea; yjkoh@cnu.ac.kr; 3 Department of Biochemistry, College of Medicine, Chungnam National University, Daejeon 35015, Republic of Korea; jipark@cnu.ac.kr; 4 Translational Immunology Institute, Chungnam National University College of Medicine, Daejeon 35015, Republic of Korea; 5 Brain Korea 21 FOUR Project for Medical Science, Chungnam National University, Daejeon 35015, Republic of Korea

**Keywords:** CD16, neurofibroma, tumor microenvironment, immunomodulation

## Abstract

Neurofibroma (NF) is a benign tumor in the peripheral nervous system, but it can infiltrate around structures and cause functional impairment and disfigurement. We incidentally found that the expression of CD16a (Fc gamma receptor IIIA) was increased in NFs compared to in non-neoplastic nerves and hypothesized that CD16 could be relevant to NF progression. We evaluated the expressions of CD16a, CD16b, CD68, TREM2, Galectin-3, S-100, and SOX10 in 38 cases of neurogenic tumors (NF, *n* = 18; atypical neurofibromatous neoplasm of uncertain biologic potential (ANNUBP), *n* = 14; and malignant peripheral nerve sheath tumor (MPNST), *n* = 6) by immunohistochemical staining. In the tumor microenvironment (TME) of the ANNUBPs, CD16a and CD16b expression levels had increased more than in the NFs or MPNSTs. CD68 and Galectin-3 expression levels in the ANNUBPs were higher than in the MPNSTs. Dual immunohistochemical staining showed an overlapping pattern for CD16a and CD68 in TME immune cells. Increased CD16a expression was detected in the ANNUBPs compared to the NFs but decreased with malignant progression. The CD16a overexpression with CD68 positivity in the ANNUBPs potentially reflects that the TME immune modulation could be associated with NF progression to an ANNUBP. Further studies should explore the role of CD16a in immunomodulation for accelerating NF growth.

## 1. Introduction

Neurofibromas (NFs) are the most common benign peripheral nerve sheath tumors. In the defective function of neurofibromin, NFs actively infiltrate around non-neoplastic structures and occur in numbers, impairing skeletal and neurological functions and cosmetic appearance and pain. NFs are subtyped into localized, diffuse, and plexiform depending on the growth pattern. According to their tumor location, NFs are classified as cutaneous and plexiform. The localized NFs usually occur sporadically as cutaneous or intraneural tumors. The diffuse NFs manifest as plaque-like cutaneous and subcutaneous tumors. The plexiform NFs present irregularly arranged superficial or deep soft tissue tumors [1]. The diffuse or plexiform form is often associated with neurofibromatosis 1 (NF1). NF1, first described by the German pathologist Friedrich von Recklinghausen, is an autosomal-dominant disorder, which refers to autosomal dominant mutations in the *NF1* tumor suppressor gene. NF1 is also caused by de novo *NF1* mutations. The *NF1* gene encodes neurofibromin protein, a GTPase-activating protein that suppresses the Ras signaling pathway. NF1 is one of the most common genetic diseases, with a frequency of occurrence of 1 in 3000 newborns without a racial, gender, or ethnic preference [2].

Peripheral nerve sheath tumors can be one of three major categories based on published pathologic literature: NF, atypical neurofibromatous neoplasm of uncertain biologic potential (ANNUBP), and malignant peripheral nerve sheath tumors (MPNSTs). Some NFs can progress to MPNSTs. The lifetime risk for malignant transformation of NF1 patients has been reported to be 8% to 16%. The category of ANNUBP has been proposed for atypical NFs displaying at least two of the following: less NF architecture, high cellularity, and mitotic figures >1/50 with <3/10 HPFs (1.5 mitoses/mm^2^). Although more reliable clinical or pathological definitions are lacking, ANNUBP has been proposed as a challenging category between the classical atypical NF and high-grade MPNST [3].

NFs are composed of neoplastic Schwann cells, fibroblasts, mast cells, macrophages, T-cells, and other stromal cells and have a predisposition to disordered cellular components in a unique collagen matrix or mucosubstances, although the cellular composition is somewhat similar to regular peripheral nerve bundles [2]. The tumor cells of NFs have been thought to be the Schwann cell lineage cells, but there is no consensus on their exact cellular origin. Moreover, the role of tumor microenvironment (TME) factors in initiating and progressing NFs has been focused. Studies on the role of individual cell types of neoplastic Schwann cells and non-neoplastic tumor microenvironment stromal cells are insufficient. Understanding the interaction between the Schwann cells and TME components is essential to developing treatment and preventing the progression of NFs [4]. Current researchers have explored mast cell infiltration in NFs as a histologic hallmark of tumorigenesis and progression [4,5]. In addition to mast cells, increased macrophage infiltration in NFs has become a focus of investigations related to NF initiation and tumor growth [6,7,8,9].

While studying the TME immune cells of NFs, we found that the expression of the Fc gamma IIIa receptor/CD16a was increased in tumor stromal cells of diffuse and plexiform NFs. This finding led us to evaluate the expression of CD16a in peripheral nerve tumor lesions along with other immune-related cytokines such as Galectin-3, a triggering receptor expressed on myeloid cells-2 (TREM2) and CD68, which are expressed during axon damage [10].

We hypothesize that the CD16a protein might be involved in NF growth associated with microenvironmental stromal cells. Hence, this study aimed to provide information on CD16a, CD16b, Galectin-3, TREM2, CD68, and SOX10 expression levels in NFs, ANNUBPs, and MPNSTs.

## 2. Materials and Methods

### 2.1. Patients and Tissue Samples

The study was approved by the Institutional Review Board of Chungnam National University Sejong Hospital (CNUSH 2022-05-004-001). The requirement for informed consent was waived because this was a retrospective immunohistochemical staining study using formalin-fixed, paraffin-embedded (FFPE) tissues. We collected samples from 38 patients who underwent surgical resection of neurogenic tumors in the subcutis or deep soft tissue between 2002 and 2022 at Chungnam National University Hospital in Daejeon, Republic of Korea, or Chungnam National University Sejong Hospital in Sejong, Republic of Korea. Depending on the growth pattern, NF refers to diffuse NFs that affect the subcutaneous and cutaneous tissue or plexiform NFs that occur in the deep soft tissue. The 38 cases of neurogenic tumors consisted of 18 cases of NF, 14 cases of ANNUBP, and 6 cases of MPNST arising in NFs. The categorization of the peripheral neurogenic tumors was based on the proposed nomenclature for a spectrum of neurofibromatous tumors (https://tumourclassification.iarc.who.int/attachment/33/92/5193 accessed on 9 June 2023) [3]. The designation ANNUBP was given to a condition with at least two of the following four features: nuclear atypia, loss of neurofibroma architecture, hypercellularity, and mitotic activity of <1.5 mitoses/mm^2^ (<3 mitotic figures per 10 HPFs) [3]. The period of freedom from recurrence (FFR) was defined as the interval months between the surgical resection date and the first recurrence or the last follow-up. Overall survival (OS) was defined as the period of months from initial surgical resection time to the date of death due to any cause. If death, recurrence, or metastasis were not confirmed, we recorded OS or FFR time based on the date the patient was last known to be alive. The clinical and pathological features of all 38 patients are summarized in Table 1.

### 2.2. Immunohistochemical Staining and Analysis

Immunohistochemical staining of the most representative whole FFPE tissue blocks was performed using a Ventana BenchMark GX automated staining instrument with the OptiView DAB IHC Detection Kit (Ventana Medical Systems, Tucson, AZ, USA). The primary antibodies used in this experiment were a rabbit monoclonal anti-human CD16a antibody (clone SP175, 1:100, ab183354; Abcam, Cambridge, UK), a mouse monoclonal anti-human CD16b antibody (clone 07, 1:100, GTX0207; GeneTex, Irvine, CA, USA), a mouse monoclonal anti-human Galectin-3 antibody (clone 9C4, ready to use, Cat#760-4256; Cell Marque, Rocklin, CA, USA), a mouse monoclonal anti-human CD68 antibody (clone PG-M1, 1:100, M0876; Dako, Glostrup, Denmark), a rabbit polyclonal anti-human TREM2 antibody (1:100, Catalog # PA5-87933; Invitrogen, Waltham, MA, USA), mouse monoclonal S-100 antibody (clone 4C4.9, ready to use, Cat# 7990-2914; Ventana, Tucson, AZ, USA), and a rabbit monoclonal anti-human SOX10 antibody (clone SP267, ready to use, Cat# 760-4968; Cell Marque, Rocklin, CA, USA). Dual immunohistochemical staining was performed using a Dako Omnis automated staining instrument with the Envision FLEX HRP MAGENTA and DAB IHC Detection Kit (Dako, Glostrup, Denmark) [11].

All the immunostained slides were digitally scanned using the PANNORAMIC^®^ 250 Flash III DX (3DHISTECH, Budapest, Hungary). Two pathologists (MKY and KHK) selected representative neoplastic areas to evaluate the expression levels quantitatively. They assessed them using the DensitoQuant module of the QuantCenter 2.2 analysis platform (3DHISTECH, Budapest, Hungary) for CD16a, CD16b, Galectin-3, CD68, and TREM2 and the NuclearQuant module for SOX10, which identified positive pixels and classified them based on stain intensity. The QuantCenter 2.2 analysis platform (3DHISTECH, Budapest, Hungary) classified positive staining intensity into four groups: 0 for negative, 1 for weak, 2 for medium, and 3 for strong. The H (histological)-scores, in the range of 0–300, were calculated by multiplying the percentage of positive pixels with the staining intensity value for each group [12].

### 2.3. Statistical Analysis

The quantitative variables were compared using the Kruskal–Wallis H test, and the Bonferroni Correction Method was used as the post hoc test. Correlations between the CD16a, CD16b, Galectin-3, CD68, and TREM2 expressions were assessed using Spearman’s correlations and the Wilcoxon signed-rank test. The Mann–Whitney U test and Pearson’s chi-squared test were used to compare the clinical values of NFs and ANNUBPs. Postoperative OS and FFR were determined using univariate Cox regression analyses. A *p*-value of less than 0.05 was considered to be statistically significant. All statistical analyses were performed using IBM SPSS version 26.0 (SPSS Inc., Chicago, IL, USA).

## 3. Results

### 3.1. CD16a and CD16b Expression Levels Were Significantly Elevated in ANNUBPs

**Figure 1:**
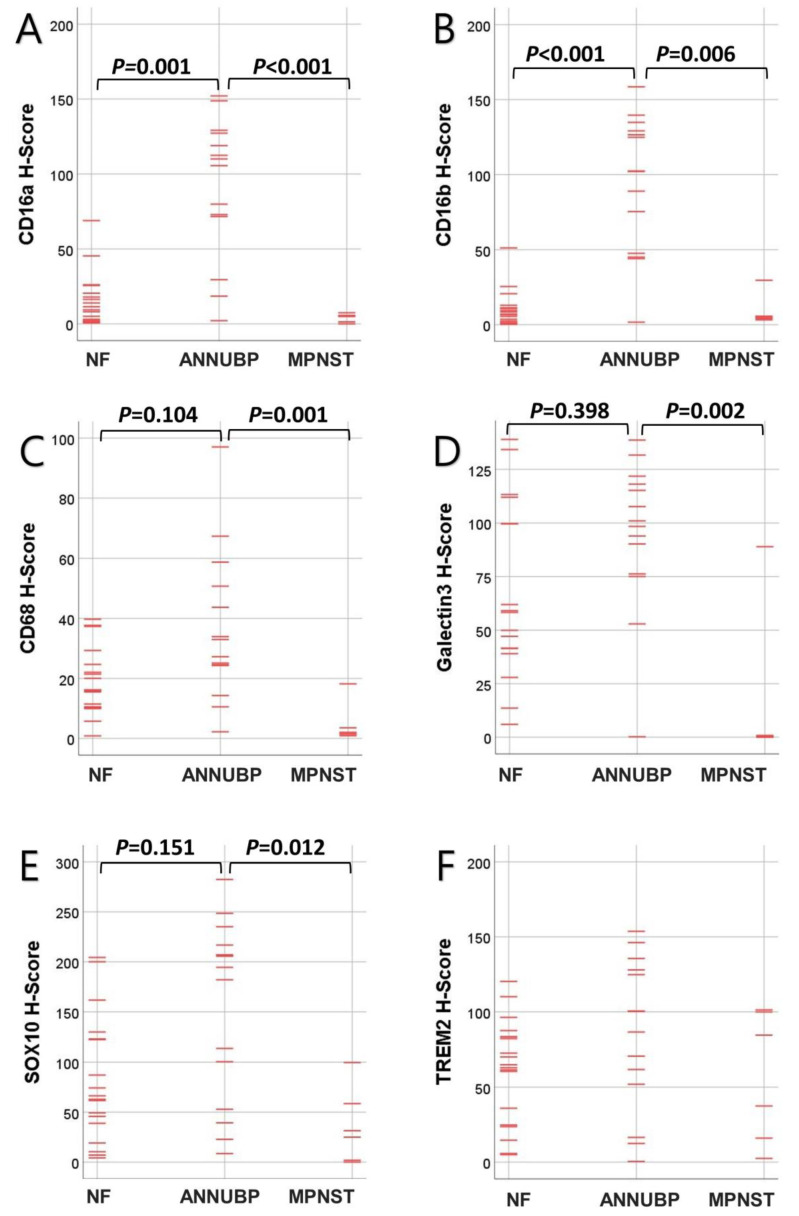
Kruskal–Wallis independent sample test scatter plots of the neurofibroma (NF), atypical neurofibromatous neoplasm of uncertain biologic potential (ANNUBP), and malignant peripheral nerve sheath tumor (MPNST) sample scores including the (**A**) CD16a, (**B**) CD16b, (**C**) CD68, (**D**) Galectin-3, (**E**) SOX10, and (**F**) TREM2 H-score results. *p-*values were calculated using a Bonferroni error correction. The TREM2 H-Score was not significantly different between NF, ANNUBP, or MPNST samples. The points are marked with red lines.

**Figure 2:**
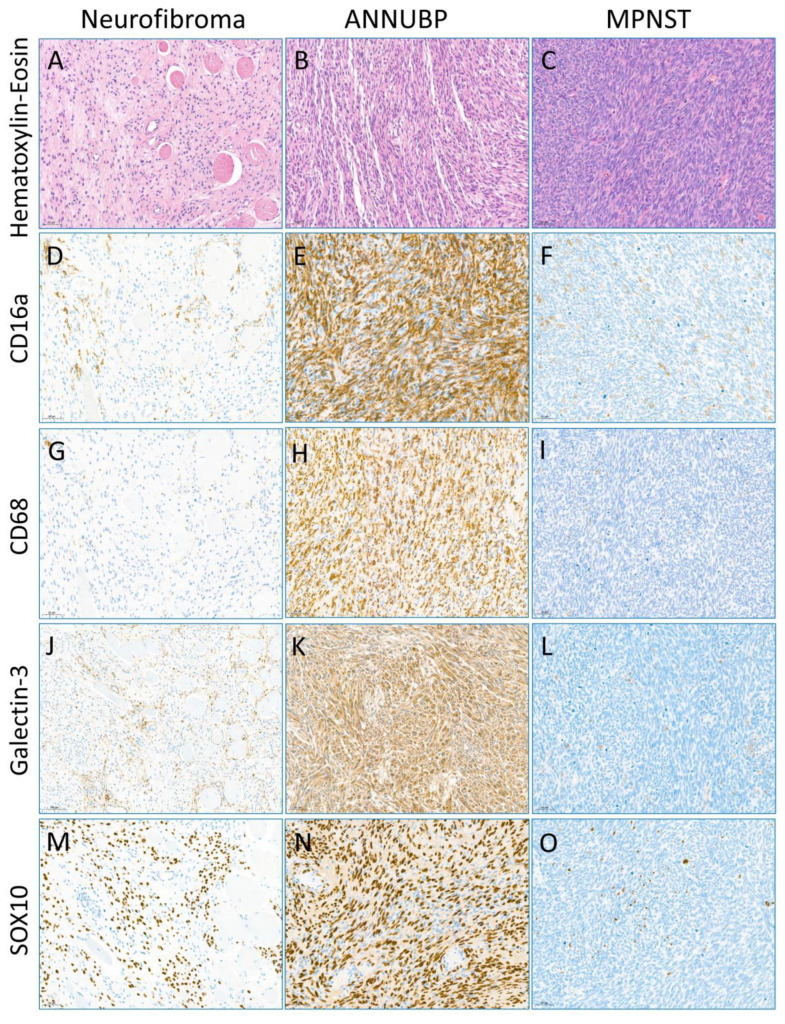
(**A**–**O**) Representative photographs of hematoxylin-eosin staining in neurofibroma (NF) (**A**), atypical neurofibromatous neoplasm of uncertain biologic potential (ANNUBP) (**B**), and malignant peripheral nerve sheath tumor (MPNST) (**C**). Representative immunohistochemical staining images of CD16a (**D**–**F**), CD68 (**G**–**I**), Galectin-3 (**J**–**L**), and SOX10 (**M**–**O**) in NF (**D**,**G**,**J**,**M**), ANNUBP (**E**,**H**,**K**,**N**), and MPNST (**F**,**I**,**L**,**O**). The immunohistochemical staining of CD16a, CD68, and Galectin-3 exhibited a cytoplasmic expression pattern. The Schwann cells showed nuclear expression of SOX10. The expression of CD16a, CD68, Galectin-3, and SOX10 is higher than in the case s of NF or MPNST (Scale bar, 50 μm, Original magnification, 200).

**Figure 3:**
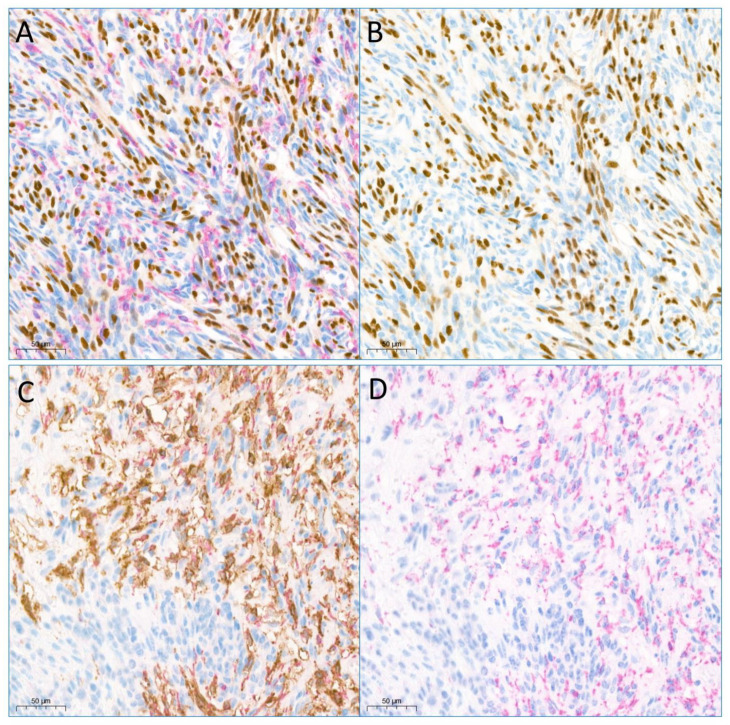
(**A**) Dual immunostaining for SOX10 and CD16a in the ANNUBP: SOX10 is expressed in the nucleus of Schwann cells (brown staining) and CD16a in the cytoplasm of tumor microenvironment immune cells (magenta staining). (**B**) Single immunostaining for SOX10 (brown color, the same section as (**A**)). (**C**) Dual staining immunohistochemistry for CD68 (magenta cytoplasmic stain) and CD16a (brown cytoplasmic stain) in the ANNUBP showed an overlapping pattern. (**D**) Single immunostaining for CD68 (magenta color, the same section as (**C**)) (Scale bar, 50 μm, Original magnification, 200).

The CD16a and CD16b expression levels in the ANNUBPs were significantly higher than in the NFs and MPNSTs (Figure 1 and Table 2). There was a significant positive correlation of the CD68 level with the CD16a and CD16b expression levels in the ANNUBPs (*p* = 0.007 and *p* = 0.003, Spearman’s correlations; *p* = 0.003 and *p* = 0.001) Wilcoxon signed-rank test. Microscopic examination revealed that in the ANNUBPs, CD16a- and CD68-expressing cells were expected to match each other but not CD16b. The cellular localization of CD16a, CD16b, and CD68 were the cytoplasm. The CD16a expression was apparently elevated in ANNUBPS and rarely showed in the non-neoplastic peripheral nerve bundles (Figure 2). We performed dual immunohistochemical staining for CD16a and SOX10 to determine if the CD16a expression was in the Schwann cells. However, the CD16a expression showed little to no in the Schwann cells but mainly in the TME immune cells (Figure 3). Dual immunohistochemical staining for CD16a and CD68 in the ANNUBPs showed an overlapping pattern for both proteins in the cytoplasm of TME immune cells (Figure 3). CD16a expression and other proteins in the 38 cases of neurogenic tumors (18 cases of NF; 14 cases of ANNUBP; and 6 cases of MPNST arising in neurofibroma) showed no association with a shorter FFR or overall survival periods in univariate Cox proportional hazard regression analysis (Table 3), nor did the 32 patients with NF or ANNUBP. The mean follow-up durations for OS and FFR were 45.55 ± 46.561 and 20.47 ± 46.561 months.

### 3.2. TREM2 Expression Was Significantly Increased along with CD16b, Galectin-3 and SOX10

TREM2 expression in all 38 cases was positively correlated with CD16b, SOX10, and Galectin-3 (*p* = 0.033, *p* = 0.003, and *p* = 0.029). The cellular localization of TREM2 and Galectin-3 was in the cytoplasm, while SOX10 showed nuclear expression. Galectin-3 expression positively correlated with CD16a, CD16b, CD68, and TREM2 in the 38 cases (*p* = 0.032, *p* = 0.044, *p* = 0.018, and *p* = 0.003) (Table 4). SOX10 and Galectin-3 expression levels in the ANNUBPs were significantly higher than in the MPNSTs (*p* = 0.012 and *p* = 0.002) (Figure 1).

## 4. Discussion

NFs are composed of neoplastic Schwann cells, fibroblasts, endothelial cells, macrophages, mast cells, other immune cells, as well as TME extracellular matrix, and studies on the role of each cell type are insufficient. The neoplastic Schwann cells and TME immune cells in NFs have different functions from normal Schwann cells or normal stromal cells and show different protein expression patterns from those of non-neoplastic peripheral nerve bundles. The growing feature of NFs is their diffuse infiltration, unlike most other benign tumors. NF progression involves diffuse Schwann cell invasion into its surroundings and unpredictable infiltrative pattern, causing a clinical dilemma. A subset of NFs develop into ANNUBPs or MPNSTs. An ANNUBP is a Schwann cell neoplasm with at least two of the following four characteristics: cytologic atypia, loss of NF architecture, hypercellularity, and mitotic index >1/50 HPFs and <3/10 HPFs. The clinicopathological and genetic definitions of ANNUBPs still need to be outlined, and correlated studies will improve our understanding of malignant transformation in NFs [3]. ANNUBPs are proposed to fall on a spectrum between classic NFs and MPNSTs. Patients with ANNUBP showed an increased risk of transition to MPNST, so ANNUBP suggests a precursor lesion of MPNST, and early detection and removal are essential for patient prognosis [13,14]. The copy number loss at the *CNKN2A/B* locus is caused by chromosome 9p21.3 deletions presented in ANNUBP. Occasionally, ANNUBP are designated as premalignant tumors, having a low risk of recurrence and no risk of metastasis. The chromosome 9p21.3 deletions, encoding *CDKN2A/B*, were not identified in NFs [15]. Inactivation of *CDKN2A* (p16^INK4A^) and its alternative reading frame p14^ARF^ was identified in MPNSTs. CNKN2A alternative reading frame (*Arf*), senescence-associated transcript, acts as a gatekeeper tumor suppressor that prevents NF progression to ANNUBP and MPNST by inducing senescence-mediated growth arrest in marked proliferating *NF1^−/−^* embryonic Schwann cell progenitors. Inactivating a single allele of *Arf* in *NF1^−/−^* Schwann cells can initiate the development of ANNUBPs and MPNSTs [16]. Further research is needed to establish and determine their biological potential.

We speculated that the tumor-promoting local microenvironment was essential for the neoplastic Schwann cells to infiltrate into the neighboring non-neoplastic tissue. In our results, CD16a was overexpressed in the TMEs of ANNUBPs and NFs but not in non-neoplastic peripheral nerve bundles, with the highest overexpression observed in ANNUBPs. Microscopically, CD16a expression was more pronounced than that of CD16b. CD16a expression was positively correlated with CD68 expression, and double-staining immunohistochemistry for CD16a and CD68 showed that the two proteins’ expression patterns overlapped. We expect CD16a to influence the TME immunomodulatory response associated with the benign peripheral nerve sheath tumor infiltration into local non-neoplastic tissue, particularly by ANNUBPs.

Schwann cells are the prevalent glial cells in the peripheral nervous system. Myelinating Schwann cells form myelin sheaths around axons to accelerate the conduction velocity of nerve impulses. The tumor cells of both cutaneous and plexiform NFs have been thought to originate from the Schwann cell lineage. However, the specific cell type of the Schwann cell lineage in NF formation is controversial. Recently published studies using genetically engineered mouse models have shown the possibility of initiating NF from the early-stage Schwann cells. The developmental stage of Schwann cell lineage characterizes the corresponding different cells, neural crest stem cells, multipotent boundary cap cells, Schwann cell precursors, immature Schwann cells, and myelinating or non-myelinating Schwann cells. The progress of the study of the tumor cell origin of NFs demonstrated the potential cellular origins. Loss of *NF1* gene in *Hoxb7* lineage-derived cells occurring before the distinct Schwann cell lineages could recapitate cutaneous or plexiform NFs. *NF1* gene inactivation in *Prss56*-positive boundary cap cells progresses to plexiform or diffuse cutaneous NFs, revealing multiple steps involved in the inflammatory processes. Several reports suggest that skin injury can accelerate the development of diffuse cutaneous NFs. *NF1-*null human-induced pluripotential stem cells in mouse SOX10-expressing cells led to NF formation [17,18,19,20]. In addition to the tumor cells, the TME on NF formation and development is crucial. The interaction between NF tumor cells and their TME is vital to promote ANNUBP or MPNST [21]. This complicated interaction needs further research.

While studying the TME immune cells of NFs, we incidentally found that CD16a expression was increased in the TME of NFs more so than in non-neoplastic peripheral nerve bundles. The fragment crystallizable γ receptors (FcγRs) have been known to express on the surface of various leukocytes and induce FcγR-dependent mechanisms against targets presented by immunoglobulin G (IgG). FcγR has binding specificity for fragment crystallizable (Fc) region of IgG. The human FcγRs are divided into two groups based on their inflammatory function: activating receptors (FcγRI/CD64, FcγRIIA/CD32a, FcγRIIC/CD32c, FcγRIIIA/CD16a, and FcγRIIIB/CD16b) and an inhibitory receptor (FcγRIIB/CD32b). The different FcγR subtypes have specific affinity for each of the IgG: FcγRI, FcγRIIB, and FcγRIIC bind to IgG1 and IgG3 equally.; FcγRIIA binds to IgG1 more strongly than IgG3.; and FcγRIIIA and FcγRIIIB bind to IgG3 more strongly than IgG1 [22,23]. CD16 exists as CD16a and CD16b, which showed a sequence homology >95%. CD16a (FcgRIIIA) is a surface receptor expressed by natural killer cells, macrophages, and monocytes. Some circulating monocytes are involved in clearing antibody-coated targets. It is involved in antibody-dependent cellular cytotoxicity and antibody-dependent cell-mediated phagocytosis and mediating direct natural killer cell cytotoxicity, whereas CD16b is expressed by neutrophils and is not involved in antibody-dependent cellular cytotoxicity [24,25]. A study showed that the homeostatic myeloid cell cluster in the murine peripheral nervous system expresses CD16 [26]. In a study of glioma, CD16-positive macrophages were observed in all the grades of gliomas but were notably lower in high-grade gliomas [27].

In this study, CD16a expression significantly increased in ANNUBPs and decreased in MPNSTs compared to NFs. For NFs to progress to ANNUBPs, CD16a expression may be required for Schwann cells to infiltrate and proliferate into the surrounding tissues; however, we predict that MPNST cells can independently invade non-neoplastic tissues without CD16a and that the CD16a expression in the ANNUBPs might be associated with neuro-inflammatory proliferative processes. We believe the TME immune cells that showed CD16a expression could be a neuro-inflammatory immune reaction involving local tissue homeostasis disruption for NF expansion into the non-neoplastic surrounding tissues.

NFs comprise tumor Schwann cells, fibroblasts, vascular endothelial cells, and various immune cells. Among the immune cells, NF-associated mast cells have been a focus of study in NF tumorigenesis. The mast cell infiltration in NF tumorigenesis is recruited by the chemoattractant stem cell factor. However, high levels of macrophages in NFs, regardless of the presence of stem cell factor, are essential in NF tumorigenesis, suggesting the role of inflammation [6]. Resident macrophages in peripheral nerves and recruited macrophages from outside are activated upon nerve injury [28,29]. In addition, macrophages are expected to have essential functions in NF development and growth because NF maintenance is impaired in macrophage-depleted environments [8,30]. The fluorescence-activated cell-sorted macrophages showed significantly different cytokine gene expression profiles in NFs compared with the non-neoplastic peripheral nervous system [9].

After facial nerve axotomy, a retrograde axonal injury model had increased phagocytic marker (CD16, CD68, and Galectin-3) and TREM2 expression, suggesting increased proliferation and phagocytosis after nerve injury [10,27,31,32,33]. TREMs are a family of activating immune receptors that regulate the inflammatory response. Based on reports of interactions between Schwann cells and TME immune cells in nerve injury or neurogenic diseases, we expected that the neuroinflammatory-related proteins (CD16, CD68, galectin-3, and TREM2) might also be specifically expressed in peripheral neurogenic tumors.

We evaluated the CD16a, CD16b, CD68, TREM2, and Galectin-3 expression levels in NF (*n* = 18), ANNUBP (*n* = 14), and MPNST (*n* = 6). Increased CD16a expression was detected in the ANNUBPs compared to the NFs, but it decreased with malignant progression. CD16a overexpression was positively correlated with CD16b and CD68 expression in the ANNUBPs, indicating that CD16a could affect the TME immune modulation of NF progression to ANNUBP or accelerate NF growth.

## 5. Conclusions

The present study shows that there was an increase in the CD16a-positive macrophage population in the TME of NFs, which may be associated with an enhanced susceptibility to ANNUBP. The CD16a overexpression in CD68-positive cells in the ANNUBPs potentially reflects that TME immune modulation is involved in NF progression to ANNUBP. Further studies should explore the role of CD16a in immunomodulation and acceleration of NF growth. More knowledge of the roles of CD16a in this process would help the development of potential therapeutic approaches for NF.

## Figures and Tables

**Table 1:** Summary of 38 patients and tissue samples.

Case	Neurofibroma (NF)	ANNUBP	MPNST	*p*
No. (total: 38)	18	14	6	
Age (mean)	54.50	56.43	47.33	0.613 *
Sex (F/M)	8/10	9/5	0/6	0.265 **
Type (diffuse/plexiform)	11/7	9/5	1/5	0.854 **

ANNUBP: atypical neurofibromatous neoplasm of uncertain biologic potential; MPNST: malignant peripheral nerve sheath tumor arising in neurofibroma; diffuse, diffuse growth involving dermis and subcutaneous tissue; plexiform, plexiform growth involving deep soft tissue; *, Mann–Whitney U test (NF vs. ANNUBP); **, Pearson’s chi-squared test (NF vs. ANNUBP).

**Table 2:** The correlation of the CD16a, CD16b, CD68, Galectin-3, SOX10, and TREM2 H-score results with clinicopathological factors in 38 patients with neurogenic tumors.

Variable (Mean)	No.	CD16a	CD16b	CD68	Galectin-3	SOX10	TREM2
Age (rho)	38	0.319	0.233	0.173	−0.101	0.365 *	0.256
Gender (M-W)	38	*p* = 0.014	*p* = 0.078	*p* = 0.121	*p* = 0.045	*p* = 0.294	*p* = 0.399
Female	17	63.92	56.03	28.90	84.80	111.24	76.97
Male	21	23.64	28.61	18.46	57.81	90.65	62.15
Diagnosis (K-W)	38	*p* < 0.001	*p* < 0.001	*p* = 0.001	*p* = 0.002	*p* = 0.011	*p* = 0.263
NF	18	15.51	10.00	18.81	69.07	81.58	60.13
ANNUBP	14	91.37	94.33	36.62	94.37	150.70	84.95
MPNST	6	4.11	8.77	4.61	15.17	36.09	56.96
Type (M-W)	38	*p* = 0.246	*p* = 0.736	*p* = 0.826	*p* = 0.018	*p* = 0.051	*p* = 0.311
diffuse	21	52.25	46.24	23.86	85.88	119.43	76.50
Plexiform	17	28.57	34.25	22.24	50.12	75.69	59.23

NF: neurofibroma; ANNUBP: atypical neurofibroma and atypical neurofibromatous neoplasm of uncertain biologic potential; MPNST: malignant peripheral nerve sheath tumor arising in neurofibroma; diffuse, diffuse growth involving dermis and subcutaneous tissue; plexiform, plexiform growth involving deep soft tissue; Rho: Spearman correlation coefficient; M-W: Mann-Whitney U test; K-W: Kruskal–Wallis test; *: *p* < 0.05.

**Table 3:** The results of univariate analysis of disease-free survival and overall survival in 38 patients with neurogenic tumors, including neurofibroma, atypical neurofibromatous neoplasm of uncertain biologic potential, and malignant peripheral nerve sheath tumor arising in neurofibroma (univariate Cox regression analysis).

	Disease-Free Survival				Overall Survival			
		95% CI				95% CI		
	HR	Lower	Upper	*p* Value	HR	Lower	Upper	*p* Value
CD16a	0.989	0.968	1.011	0.333	1.101	0.671	1.807	0.702
CD16b	1.014	0.991	1.037	0.243	0.907	0.571	1.440	0.678
CD68	0.996	0.966	1.027	0.787	1.056	0.796	1.400	0.708
Galectin3	1.003	0.991	1.016	0.608	0.928	0.621	1.386	0.714
SOX10	0.993	0.986	1.001	0.085	0.976	0.891	1.068	0.596
TREM2	1.002	0.985	1.018	0.834	1.174	0.904	1.525	0.229

HR, hazard ratio; CI, confidence interval.

**Table 4:** The correlation between CD16a, CD16b, CD68, Galectin-3, TREM2, and SOX10 H-score results in 38 patients with neurogenic tumors, including neurofibroma, atypical neurofibromatous neoplasm of uncertain biologic potential and malignant peripheral nerve sheath tumor arising in neurofibroma (Spearman’s correlations).

Spearman’s Rho		CD16a	CD16b	CD68	Galectin3	TREM2	SOX10
CD16a	Correlation coefficient	1.000	0.830 **	0.612 **	0.349 *	0.245	0.402 *
	Sig. (2-tailed)		0.000	0.000	0.032	0.138	0.012
	No.	38	38	38	38	38	38
CD16b	Correlation coefficient	0.830 **	10.000	0.733 **	0.328 *	0.347 *	0.337 *
	Sig. (2-tailed)	0.000		0.000	0.044	0.033	0.038
	No.	38	38	38	38	38	38
CD68	Correlation coefficient	0.612 **	0.733 **	1.000	0.382 *	0.306	0.373 *
	Sig. (2-tailed)	0.000	0.000		0.018	0.062	0.021
	No.	38	38	38	38	38	38
Galectin3	Correlation coefficient	0.349 *	0.328 *	0.382 *	1.000	0.468 **	0.257
	Sig. (2-tailed)	0.032	0.044	0.018		0.003	0.119
	No.	38	38	38	38	38	38
TREM2	Correlation coefficient	0.245	0.347 *	0.306	0.468 **	1.000	0.354 *
	Sig. (2-tailed)	0.138	0.033	0.062	0.003		0.029
	No.	38	38	38	38	38	38
SOX10	Correlation coefficient	0.402 *	0.337 *	0.373 *	0.257	0.354 *	1.000
	Sig. (2-tailed)	0.012	0.038	0.021	0.119	0.029	
	No.	38	38	38	38	38	38

**, Correlation is significant at the 0.01 level (2-tailed); *, Correlation is significant at the 0.05 level (2-tailed).

## Data Availability

All data generated or analyzed during this study are included in this article. Further enquiries can be directed to the corresponding author.
